# Supplementary material for: Coherent control of enhanced second-harmonic generation in a plasmonic nanocircuit using a transition metal dichalcogenide monolayer
Source: Nat Commun. 2024 Feb 29;15:1855. doi: 10.1038/s41467-024-46209-8 (PMC10904783; doi:10.1038/s41467-024-46209-8)
Supplement: Supplementary file 1 — Supplementary Information [file 41467_2024_46209_MOESM1_ESM.pdf]

Supplementary Information

Coherent control of enhanced second-harmonic generation in a plasmonic nanocircuit using a transition metal dichalcogenide monolayer

Pei-Yuan Wu[1], Wei-Qing Lee[1], Chang-Hua Liu[1,] & Chen-Bin Huang[1,*]*

Supplementary Note 1: Mathematical description of SHG from MoSe2 Monolayer 2

Supplementary Note 2: Mathematical expressions and polarization characteristics of SPP in TWTL 4

Supplementary Note 3: Optical setup 5

Supplementary Note 4: Design parameters for the plasmonic TWTL router circuit 6

Supplementary Note 5: Other routing polarization angles 7

Supplementary Note 6: Distinguishing SHG from two-photon PL 7

Supplementary References 8

Supplementary Note 1: Mathematical description of SHG from MoSe2 Monolayer

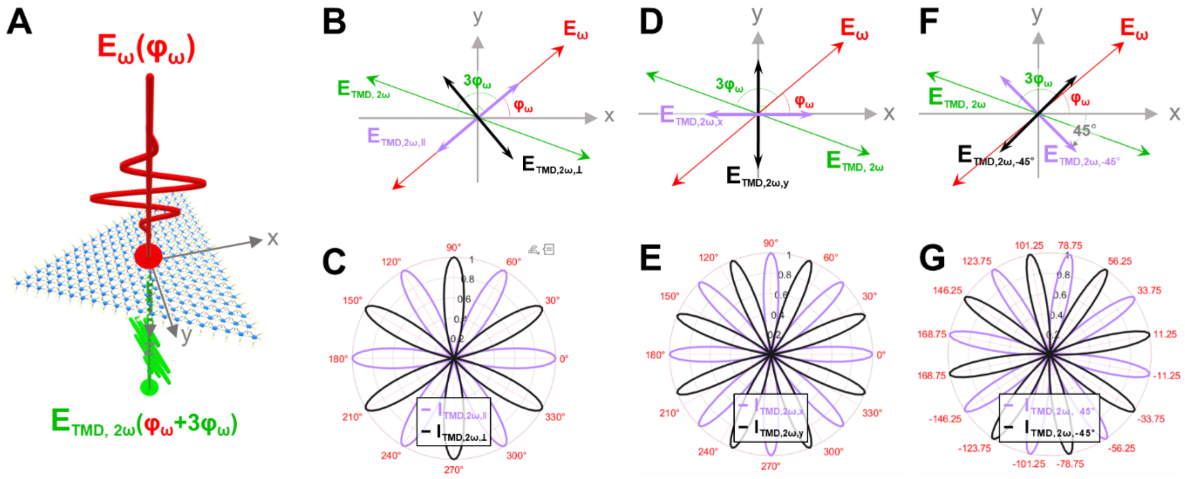

Supplementary Figure 1. Derivation mathematical of SHG from MoSe2 Monolayer. (A) shows the polarization angle SHG signal (**E$_{2\omega}$**) from $MoSe_2$ monolayer is φ_ω+3φ_ω when excited by a polarized fundamental frequency laser (**E$_\omega$**) along the armchair edge (φ_ω). (B)(C) depicted the resulting SHG in $MoSe_2$ are decomposed to two components, parallel (**E$_{TMD,2\omega,\parallel}$**) and perpendicular (**E$_{TMD,2\omega,\perp}$**), based on the fundamental laser's polarization angle. (D)(E) examined the SHG of $MoSe_2$ by utilizing a fixed polarization analyzer in both the x-direction (**E$_{TMD,2\omega,x}$**) and y-direction (**E$_{TMD,2\omega,y}$**). When a polarization analyzer is oriented at ±45°, as shown in (F)(G).

Supplementary Figure 1A illustrates the SHG signal (**E$_{2\omega}$**) with polarization angle of φ_ω+3φ_ω is generated when exciting $MoSe_2$ monolayer with fundamental frequency (**E$_\omega$**) input laser polarized at φ_ω with respect to the x-axis (along on armchair edge). This can be mathematically expressed by Supplementary Equation 1-2:

$$\mathbf{E_\omega}(\varphi_\omega) = \mathbf{x}\cos(\varphi_\omega) + \mathbf{y}\sin(\varphi_\omega)$$

$$= \mathbf{E_x} + \mathbf{E_y} \text{ --(1)}$$

$$\mathbf{E_{TMD,2\omega}}(\varphi_\omega + 3\varphi_\omega) = \mathbf{x}\cos(\varphi_\omega + 3\varphi_\omega) + \mathbf{y}\sin(\varphi_\omega + 3\varphi_\omega)$$

$$= \mathbf{E_{TMD,2\omega,x}} + \mathbf{E_{TMD,2\omega,y}} \text{ ---(2)}$$

In previous literature, to analyze the polarization dependence of SHG from TMD monolayer, the resulting SHG would be decomposed to two components[1,2]: parallel (**E$_{TMD,2\omega,\parallel}$**) and perpendicular (**E$_{TMD,2\omega,\perp}$**), according to the fundamental laser polarization

angle, as shown in Supplementary Figure 1B. The mathematical expression can be expressed by Supplementary Equation 3:

$$\mathbf{E_{TMD,2\omega}}(\varphi_\omega + 3\varphi_\omega)$$

$$= \mathbf{x}\cos(\varphi_\omega + 3\varphi_\omega) + \mathbf{y}\sin(\varphi_\omega + 3\varphi_\omega)$$

$$= \mathbf{x}\cos(\varphi_\omega)\cos(3\varphi_\omega) + \mathbf{y}\sin(\varphi_\omega)\cos(3\varphi_\omega) - \mathbf{x}\sin(\varphi_\omega)\sin(3\varphi_\omega) + \mathbf{y}\cos(\varphi_\omega)\sin(3\varphi_\omega)$$

$$= [\mathbf{x}\cos(\varphi_\omega) + \mathbf{y}\sin(\varphi_\omega)]\cdot\cos(3\varphi_\omega) + [\mathbf{y}\cos(\varphi_\omega) - \mathbf{x}\sin(\varphi_\omega)]\cdot\sin(3\varphi_\omega)$$

$$= [\mathbf{x}\cos(\varphi_\omega) + \mathbf{y}\sin(\varphi_\omega)]\cdot\cos(3\varphi_\omega) + [\mathbf{y}\sin(\varphi_\omega + 90°) + \mathbf{x}\cos(\varphi_\omega + 90°)]\cdot\sin(3\varphi_\omega)$$

$$= \mathbf{e}_{\parallel}\cos(3\varphi_\omega) + \mathbf{e}_{\perp}\sin(3\varphi_\omega)$$

$$= \mathbf{E_{TMD,2\omega,\parallel}} + \mathbf{E_{TMD,2\omega,\perp}} \text{--(3)}$$

The intensities can be expressed by Supplementary Equation 4-5, and depicted in Supplementary Figure 1C:

$$I_{TMD,2\omega,\parallel} = (\mathbf{E_{TMD,2\omega,\parallel}})^2 = \cos^2(3\varphi_\omega) \text{ --(4)}$$

$$I_{TMD,2\omega,\perp} = (\mathbf{E_{TMD,2\omega,\perp}})^2 = \sin^2(3\varphi_\omega) \text{ --(5)}$$

In this study, we analyzed the SHG of $MoSe_2$ using a polarization analyzer fixed in the x-direction ($\mathbf{E_{TMD,2\omega,x}}$) and y-direction ($\mathbf{E_{TMD,2\omega,y}}$) as shown in Supplementary Figure 1D. Then we can obtain the intensity by Supplementary Equation 6-7, and illustrate it in Supplementary Figure 1E:

$$I_{TMD,2\omega,x} = (\mathbf{E_{TMD,2\omega,x}})^2 = \cos^2(\varphi_\omega + 3\varphi_\omega) \text{ --(6)}$$

$$I_{TMD,2\omega,y} = (\mathbf{E_{TMD,2\omega,y}})^2 = \sin^2(\varphi_\omega + 3\varphi_\omega) \text{ --(7)}$$

When a polarization analyzer is oriented at ±45°, as shown in Supplementary Figure 1F, the mathematical expression is Supplementary Equation 8:

$$\mathbf{E_{TMD,2\omega}}(\varphi_\omega + 3\varphi_\omega + 45°)$$

$$= \mathbf{x}\cos(\varphi_\omega + 3\varphi_\omega + 45°) + \mathbf{y}\sin(\varphi_\omega + 3\varphi_\omega + 45°)$$

$$= \mathbf{x}\cos(\varphi_\omega + 3\varphi_\omega + 45°) + \mathbf{y}\cos(\varphi_\omega + 3\varphi_\omega - 45°)$$

$$= \mathbf{E_{TMD,2\omega,45^{\circ}}} + \mathbf{E_{TMD,2\omega,-45^{\circ}}} \text{ --(8)}$$

The intensities can be calculated by Supplementary Equation 9-10, and shown in Supplementary Figure 1G:

$$I_{TMD,2\omega,45^{\circ}} = (\mathbf{E_{TMD,2\omega,45^{\circ}}})^2 = \cos^2(\varphi_\omega + 3\varphi_\omega + 45^{\circ}) \text{ --------------------------------------(9)}$$

$$I_{TMD,2\omega,-45^{\circ}} = (\mathbf{E_{TMD,2\omega,-45^{\circ}}})^2 = \cos^2(\varphi_\omega + 3\varphi_\omega - 45^{\circ}) \text{ ------------------------------------(10)}$$

Supplementary Note 2: Mathematical expressions and polarization characteristics of SPP in TWTL

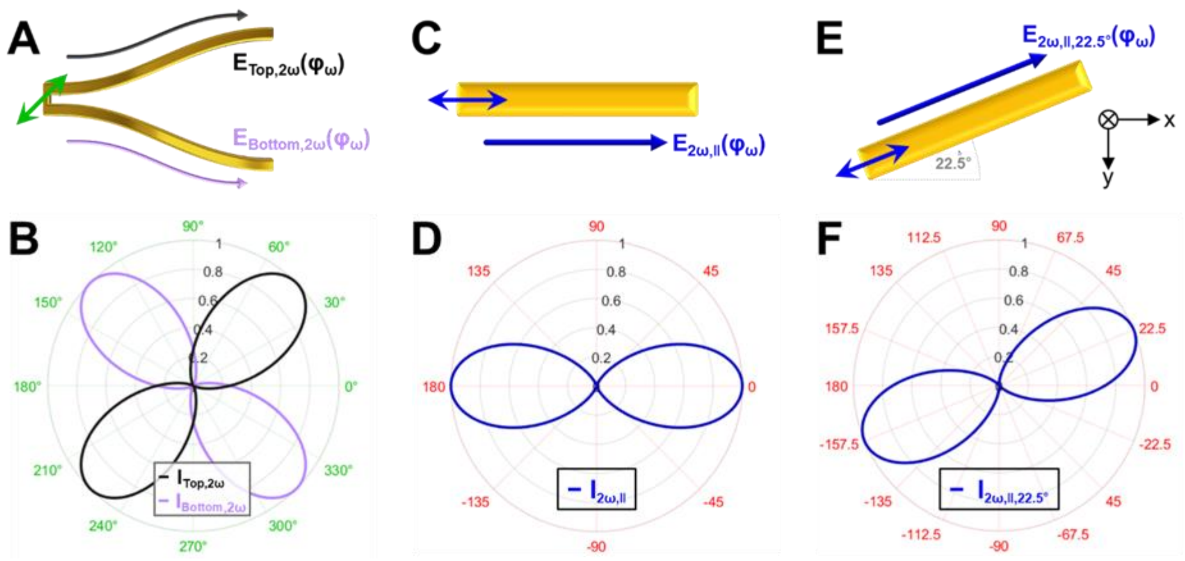

Supplementary Figure 2. Mathematical expressions and polarization characteristics of SPP in TWTL. (A)(B) schematically describes that when a linearly polarized laser excited TWTL. (C)(D) show the plasmonic single-wire transmission line can only be excited with linear polarization parallel to the wire direction. (E)(F) shown the change when the orientation of the structure is rotated.

Supplementary Figure 2A schematically describes that when a linearly polarized laser excited TWTL, the SPPs conducted on the top (**E**$_{\mathbf{Top,2\omega}}$) and bottom (**E**$_{\mathbf{Bottom,2\omega}}$) wires can be expressed as Supplementary Figure 2B, the mathematical expressions are Supplementary Equation 11 and 12:

$$\mathbf{E_{Top,2\omega}}(\varphi_\omega) = \mathbf{e_y}\left(|\cos(\varphi_\omega)|e^{j\angle\cos(\varphi_\omega)} + |\sin(\varphi_\omega)|e^{j\angle\sin(\varphi_\omega)}\right) \text{----------------------------(11)}$$

$$\mathbf{E_{Bottom,2\omega}}(\varphi_\omega) = \mathbf{e_y}\left(|\cos(\varphi_\omega)|e^{j\angle\cos(\varphi_\omega)} + |\sin(\varphi_\omega)|e^{j\angle\sin(\varphi_\omega+\pi)}\right) \text{---------------------(12)}$$

The intensities can be represented by Supplementary Equation 13-14, and depicted in Supplementary Figure 2B:

$$I_{Top,2\omega} = (\mathbf{E_{Top,2\omega}})^2 \text{---(13)}$$

$$I_{Bottom,2\omega} = (\mathbf{E_{Bottom,2\omega}})^2 \text{--(14)}$$

Supplementary Figure 2C describes that the plasmonic single-wire transmission line can only be excited when the linear polarization angle is parallel to the wire direction. The SPP ($\mathbf{E_{2\omega,\parallel}}$) on the wires can be expressed by Supplementary Equation 15:

$$\mathbf{E_{2\omega,\parallel}}(\varphi_\omega) = \mathbf{e_y}|\cos(\varphi_\omega)|e^{j\angle\cos(\varphi_\omega)} \text{---(15)}$$

The intensities can be expressed as shown in Supplementary Equation 16, and depicted in Supplementary Figure 2B:

$$I_{2\omega,\parallel} = (\mathbf{E_{2\omega,\parallel}})^2 \text{---(16)}$$

Therefore, when the orientation of the structure is rotated 22.5 degree, as shown in Supplementary Figure 2E, the polarization diagram also changes, as shown in Supplementary Figure 2F.

Supplementary Note 3: Optical setup

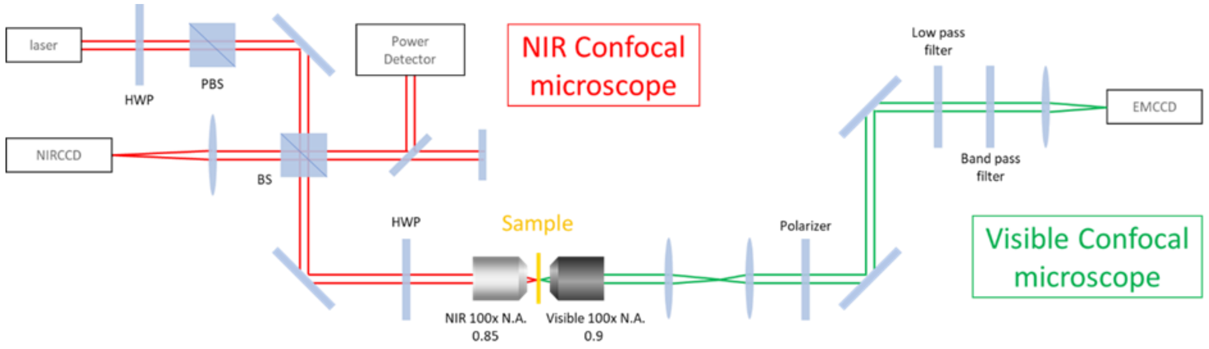

Supplementary Figure 3. A home-build two-color dual-confocal microscope. Our experiments is used a home-build two-color dual-confocal microscope. The plasmonic circulator is fabricated using focused-ion beam milling on gold film with 60 nm thickness. The fundamental laser is a 1560 nm fiber mode-locked laser (Menlo Systems T-Light). The sample is excited from substrate side and adjust the half-wave plate in front of NIR objective lens to control the polarization of fundamental laser. To check if the TMD is working, we position the polarizer behind the visible objective lens and change with fundamental laser polarization. A short pass filter (Thorlabs FESH0900) and a band-pass filter (Thorlabs FL780-10) are inserted into the detection path to ensure that the recorded images are from 780 nm signals.

Supplementary Note 4: Design parameters for the plasmonic TWTL router circuit

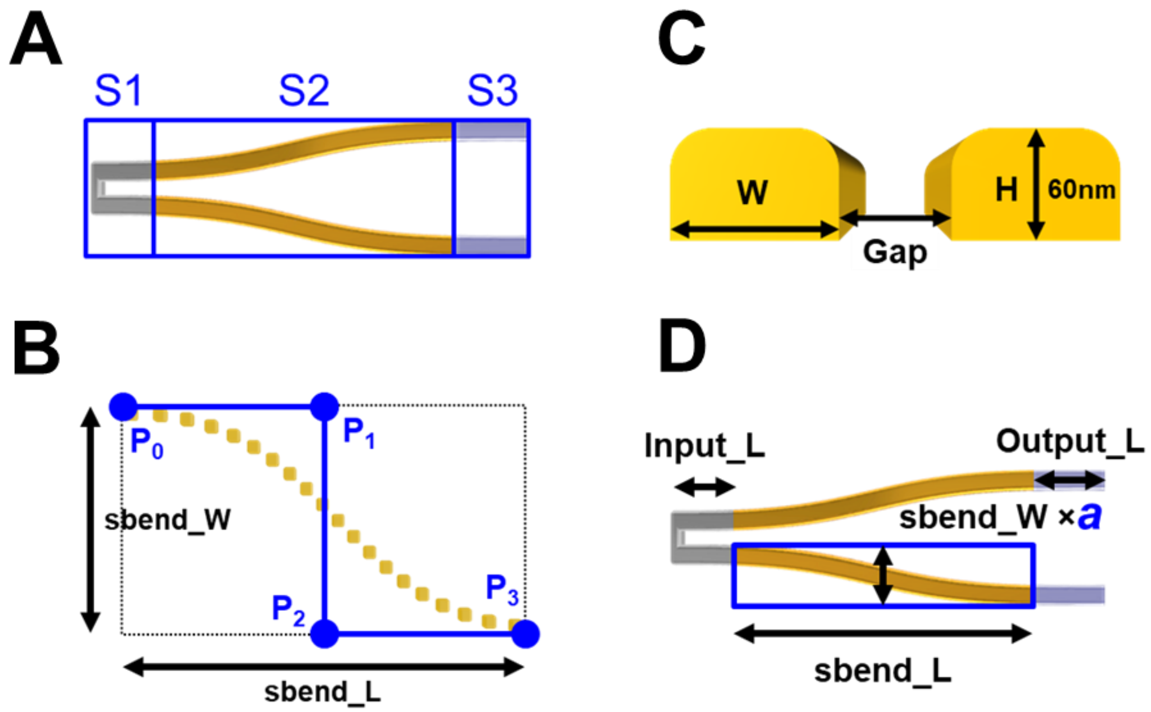

Supplementary Figure 4. Design parameters for the plasmonic TWTL router circuit. A TWTL router device is comprised of three sections: (A) section S1 is a TWTL structure that couples laser input into surface plasmon polaritons (SPP). The input source polarization provides SPP modal selectivities. The separation between the two nanowires in section S2 is gradually increased so the SPP fields are no longer coupled and complete routing. In order to reduce bending losses, section S3 is used as a buffer region for bending. (B) The cross-sectional view of the device. Basic TWTL parameters include wire width *W*, gap width *Gap*, and height *H*. In our designs we used *H* = 60 nm, *W* = 130 nm, and *Gap* = 80 nm in sections S1. (C) For section S2, the nanowires are designed according to the quadratic Bézier curve through $P(t) = (1-t)^3 P_0 + 3t(1-t)^2 P_1 + 3t^2(1-t)P_2 + t^3 P_3$. Such curve is defined through the designations of the four spatial

points (P_0~P_3). The spatial coordinates of the four points are simplified through the defining the *sbend_W* and *sbend_L* of the bended nanowire. (D) Our router circuit has values: *Input_L* = 400 nm, *Output_L* = 1500 nm, *sbend_W* = 3000 nm, *sbend_L* = 4200 nm respectively. An additional coefficients a within ranges of 0~1 provide design flexibility and optimization (here, *a* is 0.5).

Supplementary Note 5: Other routing polarization angles

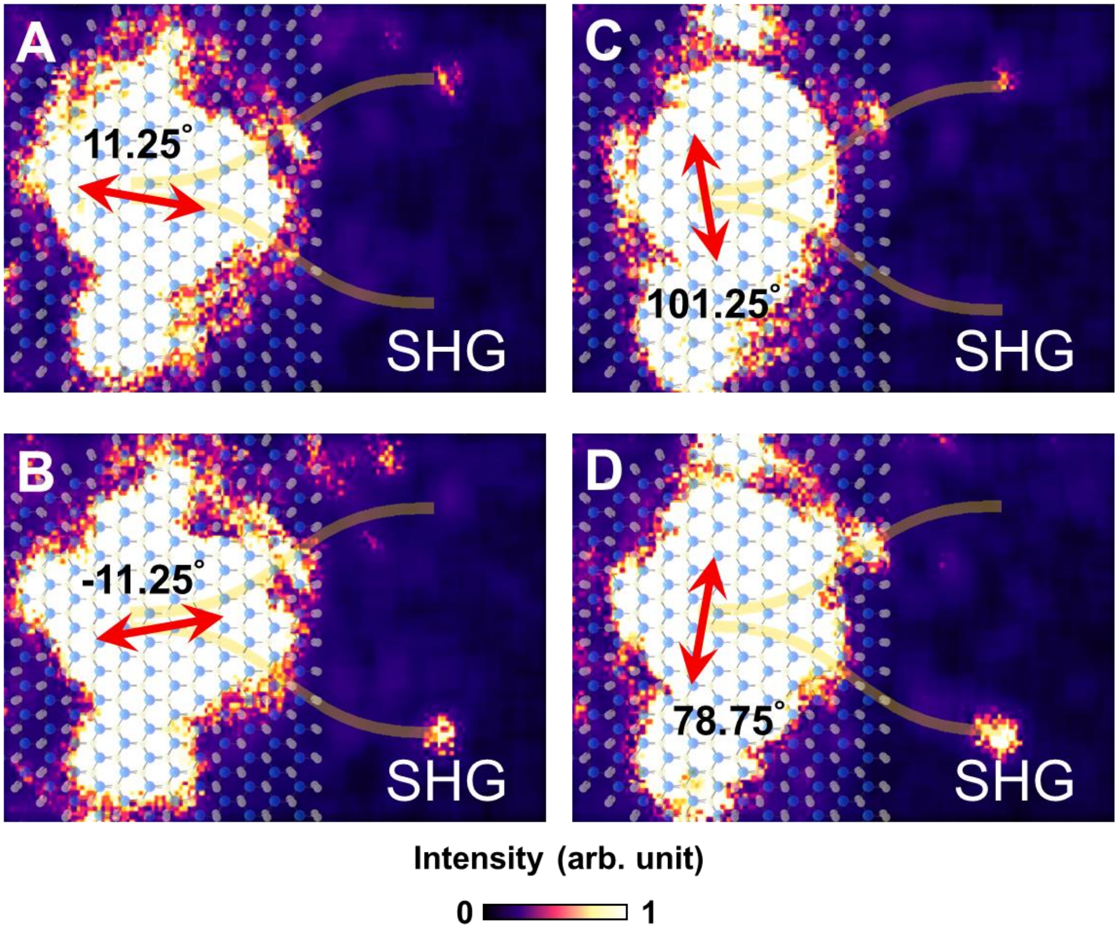

Supplementary Figure 5. Other polarization angles performance. According to the polarization dependence described in the main text, routing to the top port is expected at 11.25 degrees as well as 90 +11.25 = 101.25 degrees. On the other hand, routing to the bottom port is expected at -11.25 degrees and 90-11.25 = 78.75 degrees. Such selective routing are confirmed at (A)(B) ±11.25 degrees and (C)(D) 90 ± 11.25 degrees.

Supplementary Note 6: Distinguishing SHG from two-photon PL

Although SHG and two-photon PL from TMD have the possibility to be simultaneously excited, it is known in literature that the intensity of two-photon PL is much weaker as

compared to SHG[3,4]. SHG signal from TMDs have a unique polarization relationship[5]. On the other hand, two-photon PL, being incoherent in nature, does not possess such polarization selectivity[5]. The TMD polarization dependence studies are performed using spectral measurements (Andor Shamrock 193i with Newton CCD) to ensure that the signal routed in our nanocircuit is SHG-dominated rather than two-photon PL, as shown in Supplementary Figure 6.

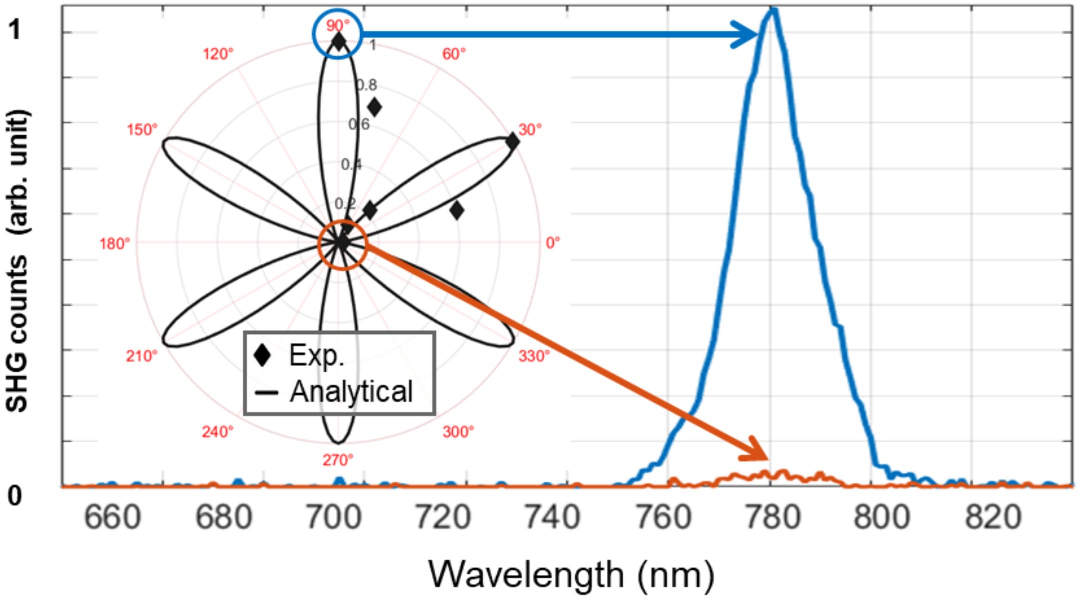

Supplementary Figure 6 Polarization dependence of SHG intensity from $MoSe_2$ monolayer through spectral measurements. The blue trace represents the spectrum of when SHG is maximum, while the orange trace denotes the two-photon PL spectrum (when the polarization analyzer minimized the SHG transmission).

Supplementary References

1 Li, Y. *et al.* Probing symmetry properties of few-layer MoS2 and h-BN by optical second-harmonic generation. *Nano letters* 13, 3329-3333 (2013).

2 Mennel, L., Paur, M. & Mueller, T. Second harmonic generation in strained transition metal dichalcogenide monolayers: MoS2, MoSe2, WS2, and WSe2. *APL Photonics* 4, 034404 (2019).

3 Granados del Águila, A. s. *et al.* Linearly polarized luminescence of atomically thin MoS2 semiconductor nanocrystals. *ACS nano* 13, 13006-13014 (2019).

4 Han, C. & Ye, J. Polarized resonant emission of monolayer WS_2 coupled with plasmonic sawtooth nanoslit array. *Nature communications* 11, 713 (2020).
5 Seyler, K. L. *et al.* Electrical control of second-harmonic generation in a WSe_2 monolayer transistor. *Nature nanotechnology* 10, 407-411 (2015).
